# Protection of coenzyme Q10 against contrast-induced acute kidney injury in male diabetic rats

**DOI:** 10.1186/s13098-021-00689-6

**Published:** 2021-06-16

**Authors:** Sheila Marques Fernandes Couto, Cassiane Dezoti da Fonseca, Mirian Watanabe, Maria de Fátima Fernandes Vattimo

**Affiliations:** 1 Laboratório Experimental de Modelos Animais (LEMA), Escola de Enfermagem da Universidade de São Paulo (EEUSP), Avenida Doutor Enéas de Carvalho Aguiar, 419, Cerqueira César, São Paulo, SP 05403-000 Brazil; 2 Escola Paulista de Enfermagem da Universidade Federal de São Paulo, São Paulo, SP Brazil; 3 Ciências da Saúde e Bem Estar (CISBEM), Centro Universitário das Faculdades Metropolitanas Unidas, São Paulo, SP Brazil

**Keywords:** Diabetes mellitus, Nephrotoxicity, Iodinated contrast, Coenzyme Q10

## Abstract

**Background:**

Diabetes mellitus (DM) is a major risk factor for contrast-induced acute kidney injury (CI-AKI). DM and CI-AKI result in oxidative damage and inflammation that can be reduced when treated with the coenzyme Q-10 (CoQ10). The aim of this study was to investigate the therapeutic potential of CoQ10 in renal function, renal hemodynamics, oxidative profile and renal histology in diabetic rats subjected to CI-AKI.

**Methods:**

Wistar rats, male, randomized into five groups: citrate: control animals received citrate buffer (streptozotocin vehicle, 0.4 mL); Tween: control animals of CoQ10 treatment received 1% Tween 80 (CoQ10 vehicle, 0.5 mL); DM: animals that received streptozotocin (60 mg/kg); DM + IC: DM animals treated with iodinated contrast (IC, 6 mL/kg); DM + IC + CoQ10: DM animals treated with CoQ10 (10 mg/kg) and that received IC (6 mL/kg). The protocols lasted 4 weeks. An evaluation was made to measure renal function, inulin clearance and serum creatinine, renal hemodynamics by renal blood flow (RBF) and renal vascular resistance (RVR), markers of oxidative stress such as urinary peroxides and nitrate, lipid peroxidation, thiols in renal tissue and renal histological analysis.

**Results:**

DM animals showed reduced renal function, which was followed by an increase inserum creatinine and significant reduction of inulin clearance and RBF. It was noticed an increase in RVR and redox imbalance with higher urinary peroxides and nitrate lipid peroxidation levels with depletion of thiols in renal tissue. IC treatment exacerbated these changes in DM + IC. CoQ10 administration ameliorated renal function, prevented hemodynamic changes and neutralized oxidative damage and progression of the histologic damage in the DM + IC + CoQ10 group.

**Conclusion:**

This study demonstrated the renoprotection properties of CoQ10 in an experimental model of risk factor of DM for CI-AKI. CoQ10 presented an antioxidant effect on the CI-AKI in male diabetic rats by improving renal function and renal hemodynamics, preserving morphology and reducing oxidative stress.

## Background

Contrast-induced acute kidney injury (CI-AKI) is the third most common cause of acute kidney injury in hospitalized patients, with a percentage of 26.6%, considering length of hospitalization and healthcare costs [1, 2]. Diabetes mellitus (DM) is one of the world’s most common chronic metabolic disorders and is associated with loss of kidney function, increasing the risk of chronic kidney disease (CKD) [3, 4]. DM is considered an important risk factor for CI-AKI. Almost 28.2% of patients that developed CI-AKI were associated with preprocedural hyperglycemia and part of them were reported to have severe reduction of glomerular filtration rate [5, 6].

Chronic hyperglycemia contributes to increased endothelin and angiotensin levels, causing intrarenal vasoconstriction, change of intrarenal blood flow, reduced pH and oxygen delivery, increased reactive oxygen species (ROS) and inflammatory cytokines [3, 7]. The increase in ROS in DM may be associated to the development of CI-AKI. Its pathogenesis is due to endothelial dysfunction, defective nitrovasodilation and cellular toxicity from the contrast media and tubular apoptosis resulting in hypoxia [2, 8]. Therefore, DM and CI-AKI share oxidative damage and inflammation mechanisms that favor oxidative stress and cytokine liberation [2, 3].

Oxidative stress is defined as an imbalance between ROS and antioxidant defenses and is responsible for the increase in mutagenic compounds, atherogenic activity and inflammatory processes [9, 10].

Coenzyme Q-10 (CoQ10) is a lipid-soluble antioxidant synthesized endogenously and a vitamin-like substance that participates in the mitochondrial respiratory chain. It has been evidenced in previous studies to play an antioxidant and anti-inflammatory role [4, 11, 12]. As an intracellular antioxidant, the efficiency of CoQ10 is due to its intramembranous localization, effective reduction/reactivation of a number of cellular systems and abundant distribution. All of these properties contribute to the protection of phospholipids and membrane proteins from oxidative damage. CoQ10 also inhibits both the initiation and propagation of lipid and protein oxidation. It has also been demonstrated to have anti-inflammatory effects participating in the modulation of inflammatory cytokines and transcription factors [12–14]. CoQ10 supplementation has already shown neuroprotective activities in animal models of diabetic nephropathy and cisplatin nephrotoxicity [15, 16].

Thus, the aim of this study was to investigate the therapeutic potential of CoQ10 in renal function, renal hemodynamics, oxidative profile and renal histology in diabetic rats submitted to the CI-AKI model.

## Methods

### Animals

Adult male Wistar rats (weighing 250–290 g) were used. The animals were acquired from the Institute of Biomedical Sciences at the University of Sao Paulo and were housed at Experimental Laboratory of Animal Models (LEMA) at the School of Nursing, University of Sao Paulo, in a room at a controlled temperature (25 ºC/77 ºF) on alternating light/dark cycles, and had free access to water and rat chow. The study was approved by the Ethical Committee of Experimental Animals, University of Sao Paulo (CEEA, protocol no. 055/15).

### Streptozotocin-induced diabetes mellitus model

The animals received 60 mg/kg of Streptozotocin (STZ, Chemical Company, USA), diluted in 0.4 mL citrate buffer (0.1 mol/L; pH 4.5), intravenously (i.v.) into tail vein, for DM type 1 induction. Blood glucose levels were measured 48 h later to confirm hyperglycemia (Accu-Chek, Roche, USA) and considered diabetic the animals that showed blood glucose level higher than 250 mg/dL [17].

### Iodinated contrast induced CI-AKI model

The animals received 6 mL/kg of iodinated contrast (IC, meglumine ioxithalamate and sodium) intraperitoneally (i.p.), single dose [18].

### Coenzyme Q10 administration

Animals were subjected to administration of 10 mg/kg of CoQ10 (Sigma Chemical Company, USA) i.p., dissolved in 0.5 mL of 1% Tween 80 solution for 6 days [19].

### Experimental groups

Citrate (n = 7): control group of chronic DM model, rats that received 0.5 mL of citrate buffer (STZ vehicle), i.v. into tail vein, single dose on the 1st day;

Tween: control group of CoQ10 treatment; rats received 0.5 mL of 1% Tween 80 (CoQ10 vehicle), i.p., for 6 days, starting on the 22nd day of the experiment.

Diabetes mellitus (DM, n = 7): rats received 60 mg/kg of STZ diluted in 0.5 mL of citrate buffer i.v. into tail vein; single dose on the 1st day;

Diabetes mellitus + iodinated contrast (DM + IC, n = 7): DM rats that received 6 mL/Kg of IC intraperitoneal (i.p.); single dose on the 26th day.

Diabetes mellitus + iodinated contrast + coenzyme Q10 (DM + IC + CoQ10, n = 7): DM rats that received CoQ10, i.p., diluted in 0.5 mL of 1% Tween 80. Treatment with CoQ10 started on the 22nd day of the experimental protocol. There were four preconditioning days, followed by a dose administered on the same day as IC administration and the last dose on the following day.

### Procedures and timing of experimental protocols

**Fig. 1:**
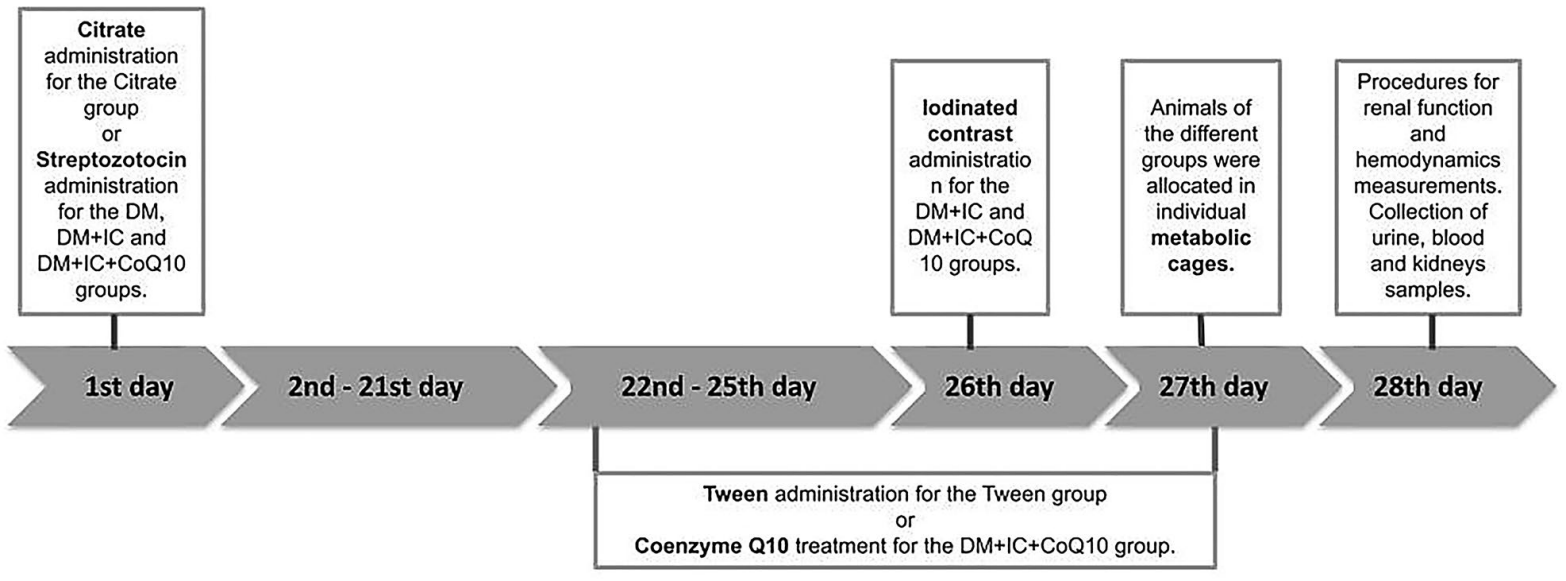
Timeline of the experiments

All protocols of experimental groups lasted 4 weeks (28 days) and the timeline of the experiments is illustrated in Fig. 1. STZ or Citrate was administered on the 1st day of the experimental protocol. The treatment with CoQ10 or Tween occurred during the 22nd to the 27th day, while the CI administration was performed on the 26th day of the experimental protocol.

Animals were allocated in individual metabolic cages on the 27th day, for 24 h, for collection of urine and determination of urinary flow on the 28th day of the protocol, the rats were anesthetized with 10 mg/kg xylazine and 90 mg/kg ketamine i.p., and underwent surgical procedure for renal function and hemodynamics measurements. After that, a blood sample was collected through a puncture of the abdominal aorta. Finally, the animals were submitted to euthanasia according to guidelines for animal experimentation and removal of kidneys for thiols assay and histological analysis.

### Renal function measurement

Inulin clearance (mL/min): renal function was evaluated based on inulin *clearance*. Inulin was dissolved in saline and injected in the right jugular vein using a catheter (polyethylene tube PE-60). A loading dose of 100 mg/kg body wt of inulin solution of 20 mg/mL was followed by a continuous infusion of 0.04 mL/min of inulin solution of 6 mg/mL. After a 30 min equilibration period, three urine collections were made through the bladder catheter and two blood samples were obtained at the carotid catheter. The serum and urinary inulin were measured using the Anthrone method [20, 21].

Serum creatinine (Cr) (mg/dL): was measured using the Jaffé method. For further analysis, the serum was deproteinized using 500 µL of the sample diluted in 1500 µL of distilled water and were deproteinized by adding 250 µL of 5% sodium tungstate and 250 µL of sulfuric acid 0.75 N. After homogenization, the solution was centrifuged at 4500*g* for 10 min. For dosing purposes, 200 µL of the supernatant were removed and added to 300 µL of alkaline picrate 0.040 N and 300 µL of sodium hydroxide 0.75 N. The mixture was then incubated for 20 min at room temperature and the absorbance was read at 520 nm by the spectrophotometer. The results were expressed in mg/dL [22].

### Renal hemodynamic measurement

Renal blood flow: was measured by an ultrasonic flowmeter (T402, Transonic Systems Inc., USA) placed around the isolated renal artery [20].

Renal vascular resistance: mean arterial blood pressure (MAP) and renal blood flow (RBF) were measured by a catheter inserted into the left carotid (polyethylene tube PE-60) and the renal vascular resistance (RVR) was calculated with the formula RVR = MAP/RBF [20].

### Oxidative profile

Urinary peroxides: urinary peroxides were determined by the ferrous oxidation of xylenol orange version 2 (FOX-2) method. Ferrous iron was oxidized to ferric iron by peroxides contained in the samples. Xylenol orange shows a high selectivity for the Fe^3+^ ion, producing a bluish purple complex that can be measured at 560 nm (ε = 4.3 × 10^4^ M^−1^ cm^−1^). 100 µL of urine samples were mixed with 900 µL of FOX-2 reagent (90 mL methanol; 10 mL double distilled water; 100 µM xylenol orange, 4 mM butylated hydroxytoluene, 25 mM sulfuric acid and 250 µM ferrous ammonium sulfate), and were vortexed and incubated for 30 min at room temperature. The solutions were then centrifuged at 400*g* for 10 min at 4 °C and the absorbance was read at 560 nm. The values were expressed in nmol urinary peroxides/g urinary Cr [23].

Urinary nitrate: nitrate is one of the stable metabolites of nitric oxide and was determined in urine by the Griess method. A mixture of 1% sulfanilamide (in 5% H_3_PO_4_) and 0.1% naphthylethylenediamine solution (Sigma-Aldrich, Saint Louis, USA) was added to the urine samples, and the absorbance was measured at 546 nm using a GENESYS 2 spectrophotometer (Spectronic Instruments, Rochester, USA). The nitrate was then estimated from a standard calibration curve for sodium nitrate from external sources. The data was reported in nmol nitrate/mg urinary Cr [24].

Urinary thiobarbituric acid reactive substances (TBARS): in order to assess lipid peroxidation, the levels of the peroxidation product malondialdehyde were determined by measuring thiobarbituric acid-reactive substances (TBARS). TBARS were measured in urine. For that quantification 0.4 mL of a urine sample was diluted in 0.6 mL water and was immediately added to a reaction mixture consisting of 1 mL 17.5% trichloroacetic acid and 1 mL 0.6% thiobarbituric acid. This mixture was heated in a double boiler at 95 °C for 20 min to promote the reaction with thiobarbituric acid. The solution was then removed from the double boiler and cooled in ice, followed by the addition of 1.0 mL 70% TCA. The solution was homogenized and incubated for 20 min at room temperature. After that, the solution was centrifuged at 1600*g* for 15 min and the absorbance was read in a spectrophotometer at 534 nm. Concentration of lipid peroxidation products was calculated as the malondialdehyde equivalent using a molar extinction coefficient of 1.56 × 10^5^ M^−1^ cm^−1^. Urinary levels of TBARS were expressed as nmol/g urinary Cr [25].

Soluble non-protein thiols in renal tissue: non-protein soluble thiols in renal tissue were assessed in homogenized tissue in 1 mL of a solution containing 10 mM of sodium acetate, 0.5% Tween 20 and 100 μM of diethylenetriaminepentaacetic acid (DTPA, pH 6.5). The homogenates were centrifuged at 4500*g* for 10 min at 4 °C. One aliquot was reserved for immediate measurement of total protein content using the Bradford assay method, and the second aliquot was precipitated with 20% trichloroacetic acid (1:1) to measure total thiol content. After deproteinized, the samples were homogenized in 100 μL of a solution containing 100 mM of Tris buffer (pH 8.0) and 5,5-dithio-bis-2-nitrobenzoic acid (DTNB, Ellman’s reagent). After 10 min at room temperature, the amount of thiols was determined based on the mean absorbance at 412 nm (ε = 13.6 × 10^3^ M^−1^ cm^−1^). The amount of soluble thiols was then corrected for the total protein content and was expressed as nmol/mg total protein [26, 27].

Urinary Cr by Jaffé method was used to normalize oxidative parameters [22].

### Histological analysis

Tubulointerstitial damage: sections from the left kidney were fixed in a methacarn solution (methanol:chloroform:acetic acid, 6:3:1) for histological analyses. Kidney tissue embedded in paraffin was sectioned at 4 μm and the paraffin sections of fixed kidneys were stained with hematoxylin and eosin for analysis via light microscopy. Five slices of each group were observed and tubulointerstitial damage was evaluated at 20 grid fields (0.245 mm^2^ each; magnification, × 400) per animal. Damage was scored by calculating the percentage of tubules that presented atrophy, epithelial edema, tubular lumen dilatation, vacuolar degeneration or presence of an inflammatory cell infiltrate, as follows: 0, < 5%; I, 5–25%; II, 26–50%; III, 51–75%; and IV, > 75%. To minimize bias, the observer was blinded to the treatment groups [20].

### Statistical analysis

The results are reported as the mean ± standard error (SEM). The analysis of variance by One Way ANOVA and post hoc Newman-Keuls test were used for comparisons of groups. Statistical significance was defined at p < 0.05. All statistical analyses were performed in Graph-Pad Prism version-7 for Windows^®^.

## Results

### Effect of CoQ10 treatment on renal function

**Fig. 2:**
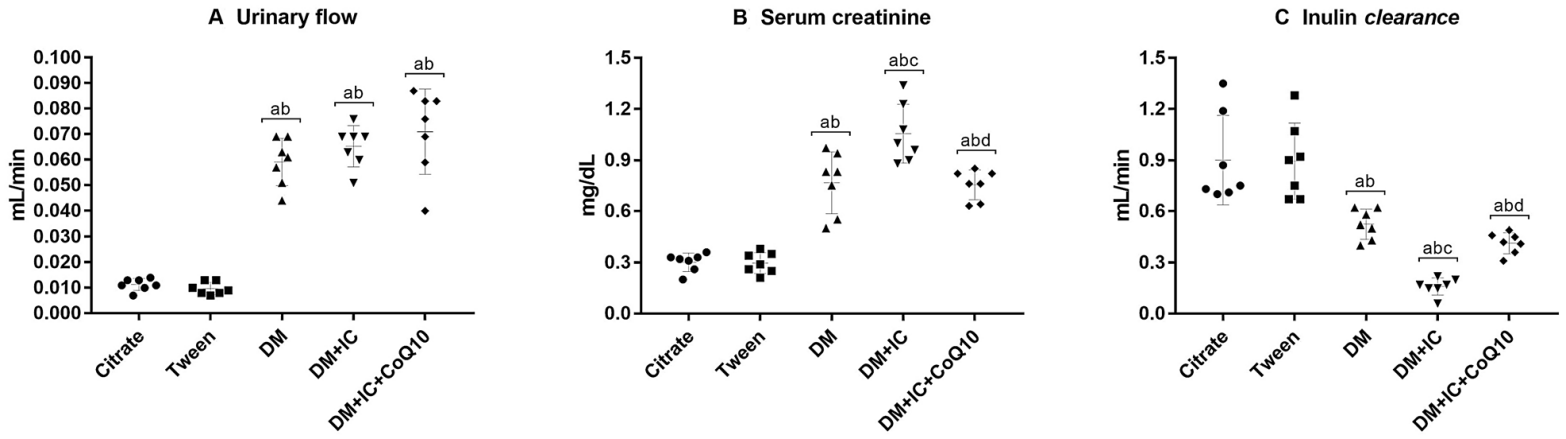
Renal function: Urinary flow (**A**), serum creatinine (**B**), inulin *clearance* (**C**). ^a^p < 0.05 vs Citrate; ^b^p < 0.05 vs Tween; ^c^p < 0.05 vs DM, ^d^p < 0.05 vs DM + IC

The results showing the effect of CoQ10 on renal function after injury are represented in Fig. 2. Rats submitted to DM showed a significant increase in urinary flow, serum Cr and a decrease in inulin clearance. DM + IC group resulted in additional elevation in serum Cr and a reduction in inulin clearance compared to the DM group, these parameters were altered by the CoQ10 treatment, which significantly decreased serum Cr and improved inulin clearance as is shown in the DM + IC + CoQ10 group.

### Effect of CoQ10 treatment on hemodynamic parameters

**Fig. 3:**
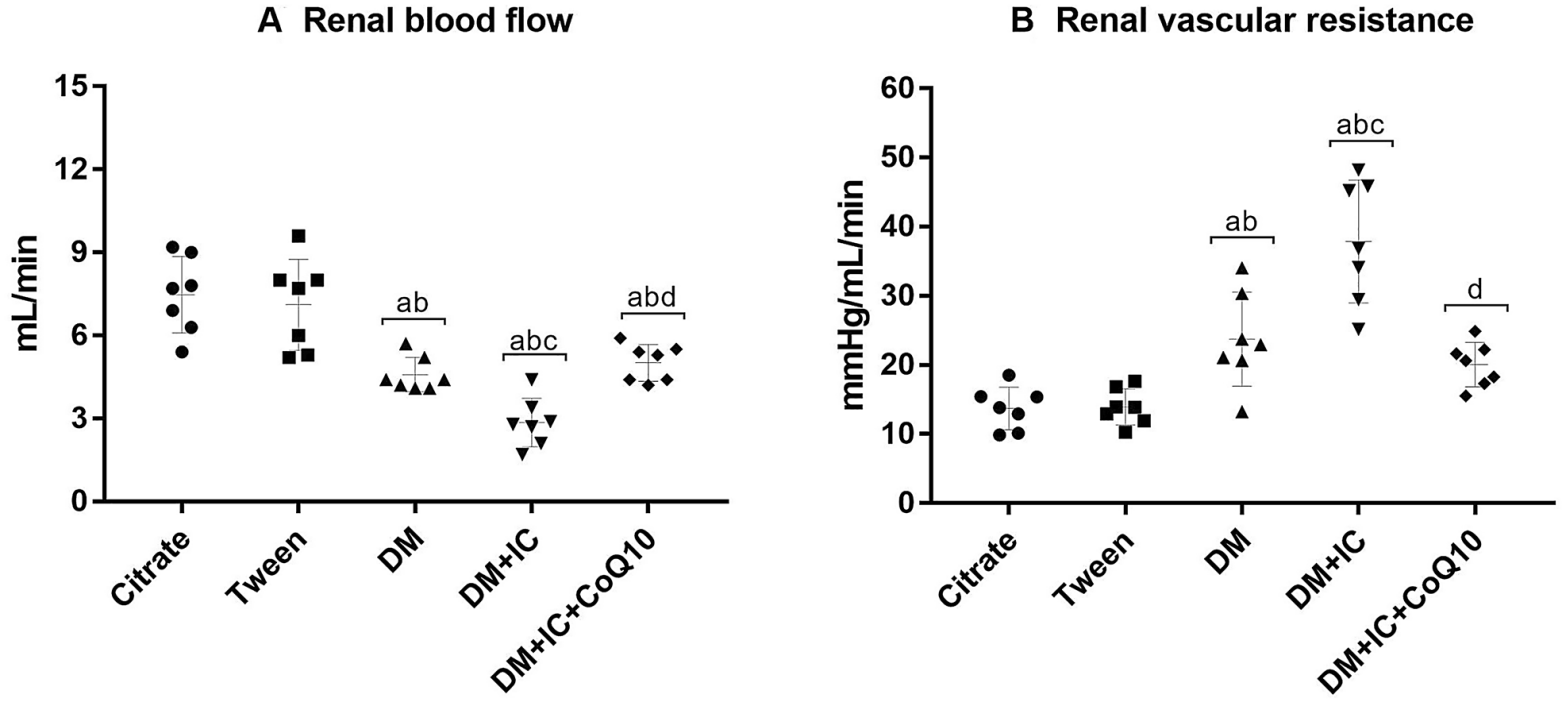
Renal hemodynamic: renal blood flow (**A**), renal vascular resistance (**B**). ^a^p < 0.05 vs Citrate; ^b^p < 0.05 vs Tween; ^c^p < 0.05 vs DM, ^d^p < 0.05 vs DM + IC

Data illustrated in Fig. 3 show the effect of CoQ10 in renal hemodynamics. A significant elevation of RVR and reduction of RBF in the DM groups were observed. These changes were exacerbated in the DM + IC group. As shown in Fig. 3, the treatment with CoQ10 significantly increased RBF and decreased RVR in the DM + IC + CoQ10 group.

### Effects of CoQ10 treatment on oxidative profile

**Fig. 4:**
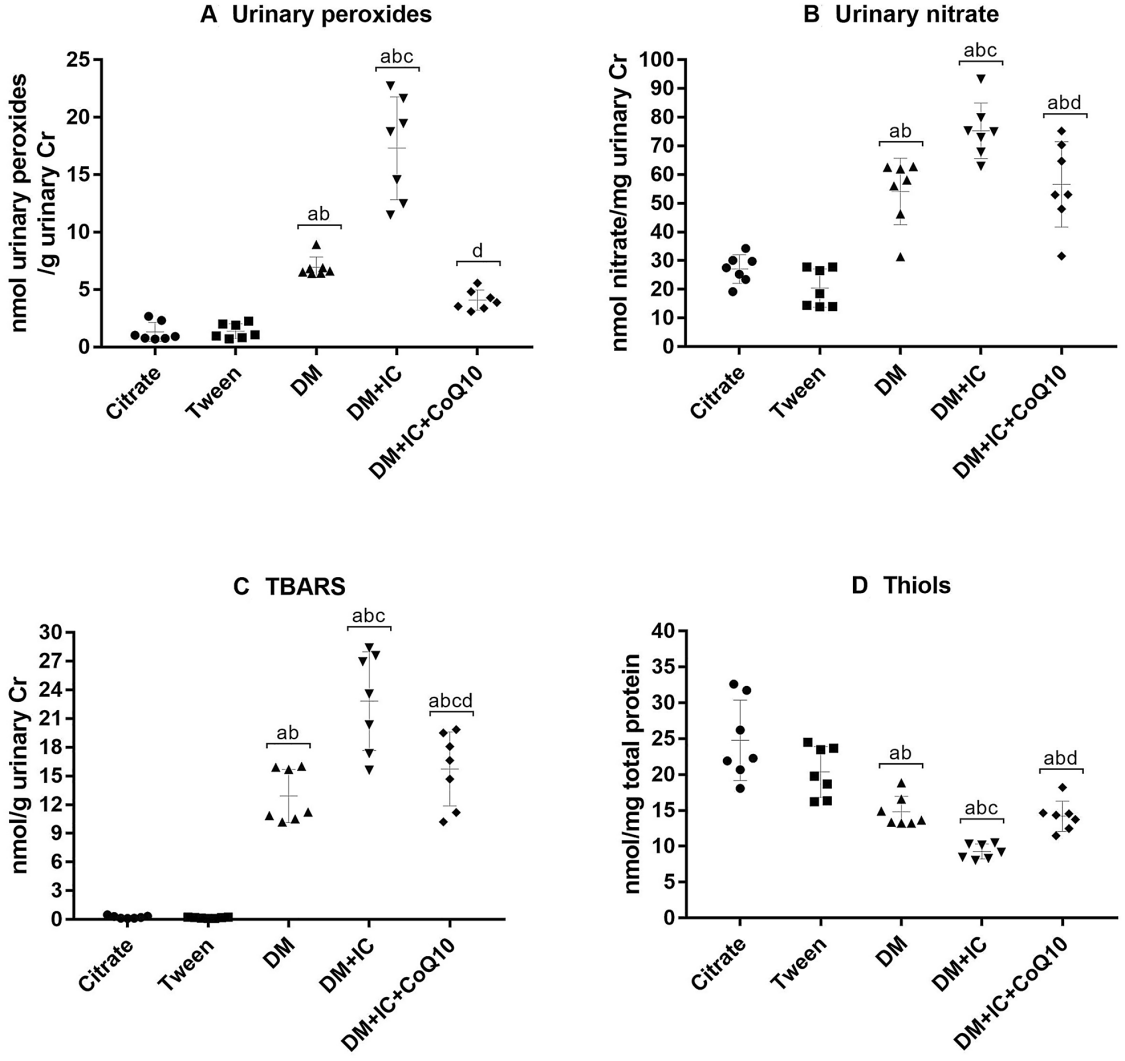
Oxidative profile: urinary peroxides (**A**), urinary nitrato (**B**), TBARS (**C**), thiols (**D**). ^a^p < 0.05 vs citrate; ^b^p < 0.05 vs Tween; ^c^p < 0.05 vs DM, ^d^p < 0.05 vs DM + IC

Figure 4 summarizes the oxidative profile. DM groups showed a significant increase in concentrations of oxidative metabolites and a reduction of soluble non-protein thiols levels in renal tissue. The redox imbalance findings were more pronounced in the diabetic group that was treated with IC, DM + IC, compared to DM. Treatment with CoQ10 reduced oxidative stress, as demonstrated by decreased urinary peroxides, nitrate and TBARS excretion in DM + IC + CoQ10 group. Furthermore, CoQ10 significantly preserved antioxidant capacity, expressed by increased soluble non-protein thiols in renal tissue.

### Histological analysis

**Fig. 5:**
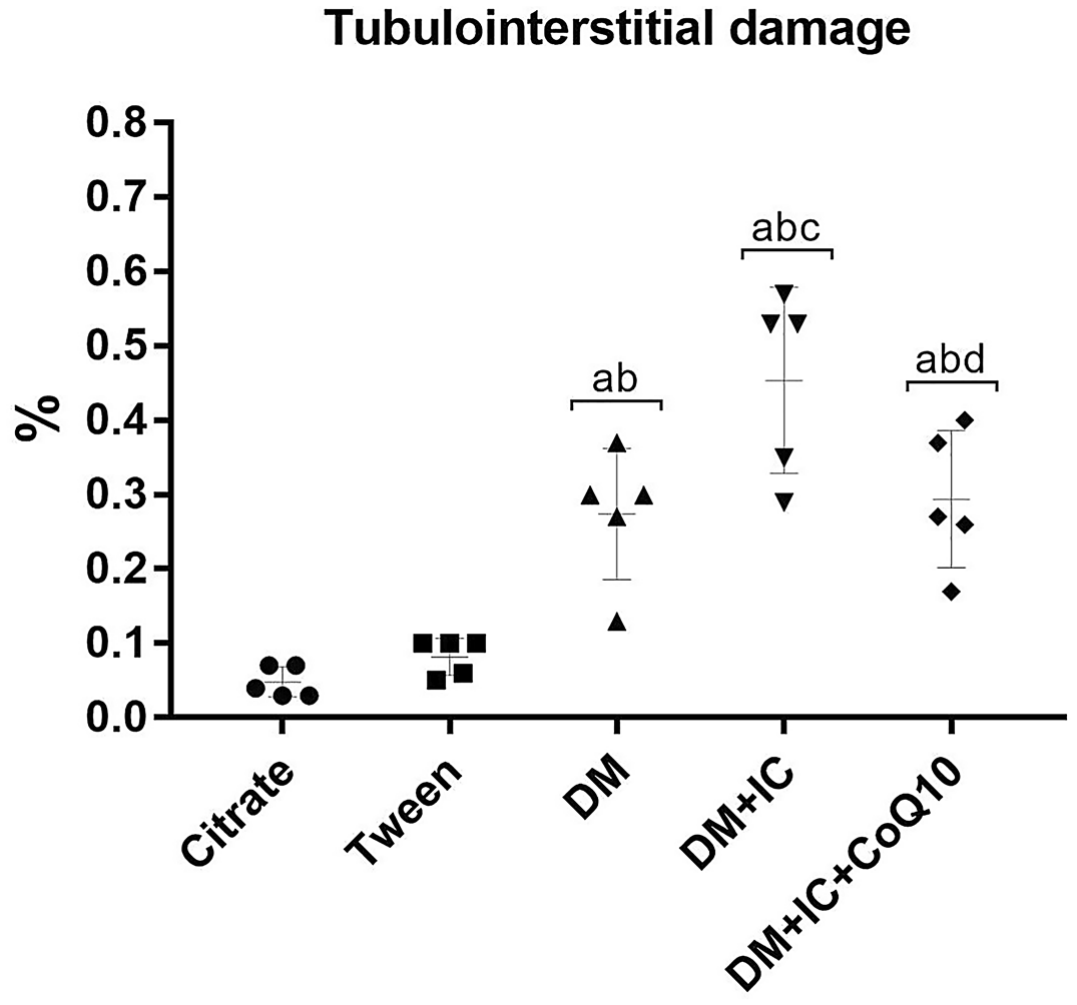
Tubulointerstitial damage. ^a^p < 0.05 vs citrate; ^b^p < 0.05 vs Tween; ^c^p < 0.05 vs DM, ^d^p < 0.05 vs DM + IC

**Fig. 6:**
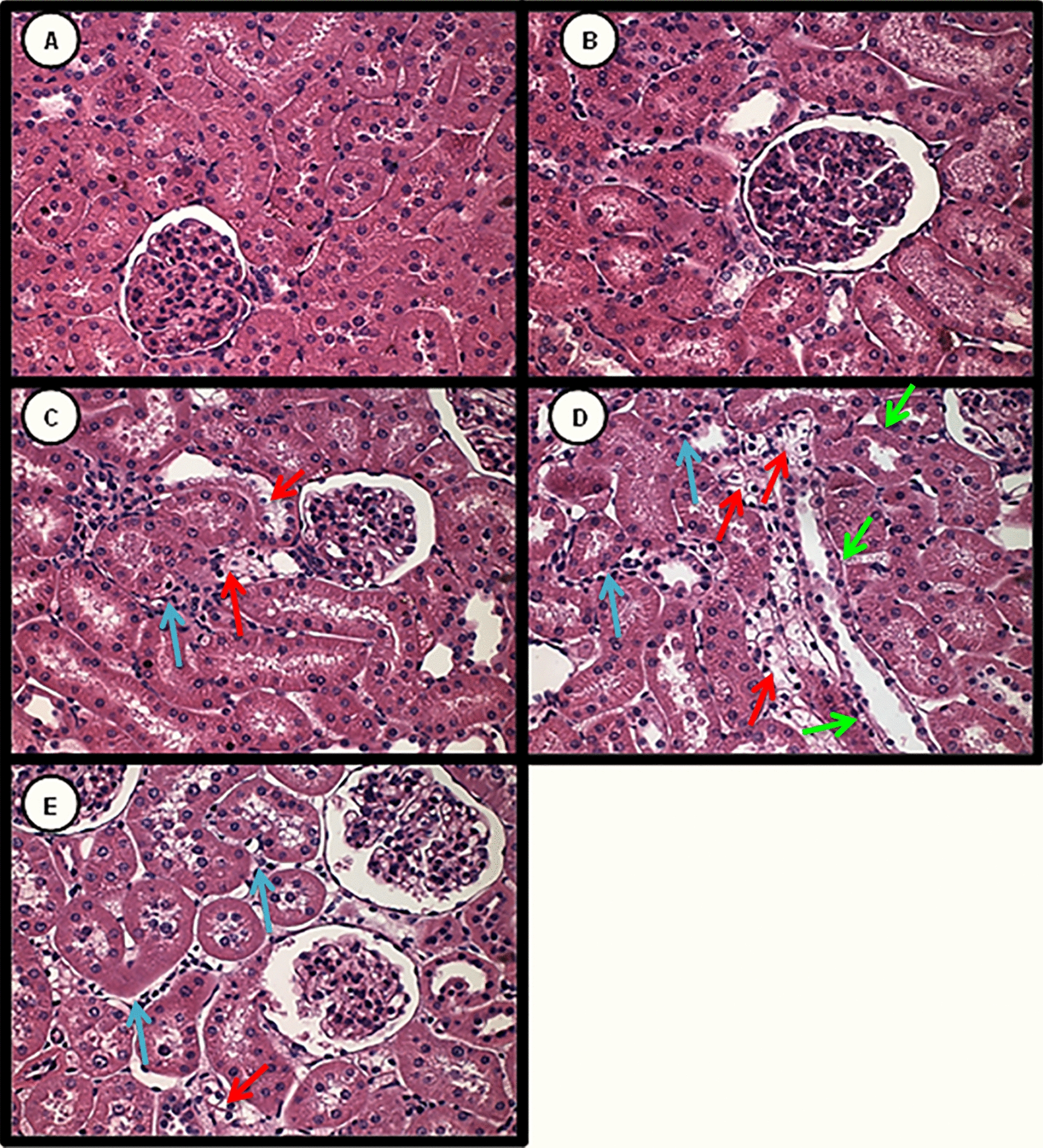
Tubulointerstitial damage (magnification, × 400) of the groups: citrate (**A**), Tween (**B**), DM (**C**), DM + IC (**D**), DM + IC + CoQ10 (**E**). Arrows: Red—epithelial edema, green—flattening of tubular cells, blue—inflammatory interstitial infiltration. Source: Fernandes SM. The role of coenzyme Q-10 in acute contrast-induced renal injury in diabetic rats [dissertation]. São Paulo: EEUSP: 2016

As shown in Figs. 5 and 6, all groups showed slight changes with impairment of < 5% of the tissue focal areas. Histological changes in the DM group were significantly higher compared to Citrate and Tween groups. Figure 6C shows DM resulted mainly in a discrete edema and increased interstitial area. The DM + IC group showed a significant increase in the tubulointerstitial lesion area compared to the DM group. After IC (Fig. 6D), kidneys presented tubulointerstitial injury characterized by edema, flattening of tubular cells and diffuse inflammatory interstitial infiltration. Treatment with CoQ10 showed significant reduction in the extension area of the tubulointerstitial lesion compared to DM + IC as illustrated in Fig. 6E of renal histological analysis.

## Discussion

The present study evaluated the participation of oxidative stress in the pathophysiology of CI-AKI with DM as a risk factor and investigated the role of CoQ10 as a possible treatment for this pathology. Oxidative stress and inflammation status are correlated in the prognosis of CI-AKI in DM, therefore, the investigation of antioxidants alternatives that promote renoprotection is still of great importance.

The development of CI-AKI in DM was evidenced in this study with dysfunctions in renal function, renal hemodynamics and oxidative damage. Clinically, CI-AKI is defined by an increase in serum creatinine ≥ 0.5 mg/dL or 25% increase of serum creatinine from the baseline value at 48 h after contrast media administration [28].

In a recent publication authors have demonstrated the effect of physical training as an antioxidant and hemodynamic modulator on IC nephrotoxicity in DM in experimental settings, highlighting this nonpharmacological therapy as a potential modifier of risk of CI-AKI in DM [29]. The present study identified CoQ10 as a promising pharmacological therapy for renoprotection in diabetic male rats submitted to IC treatment.

CoQ10 has demonstrated high therapeutic potential due to its antioxidant and anti-inflammatory properties in many injury models, including studies of nephrotoxicity by cisplatin and cyclosporine [16, 30–32]. Our results highlighted the role of CoQ10 in the modulation of pathophysiological processes induced by nephrotoxicity of IC, showing that the treatment with CoQ10 exerted a protective effect on the renal function of diabetic animals submitted to CI-AKI. The renoprotective effect was evidenced by the increase in inulin clearance and decrease in serum creatinine in DM animals that received IC and treatment with CoQ10.

Increased RVR and decreased RBF in DM after IC, as observed in this study, can be attributed to vasoconstriction due to viscosity and osmolarity of contrast media. The vasoconstriction contributes to hypoxia and development of oxidative injury that can culminate into endothelial dysfunction [4, 26]. Additionally, DM is associated with development of hypoxia inducible factors (HIF) that enhance activity of renin-angiotensin systems and may also intensify the vasoconstriction via endothelin synthesis and increase effect of adenosine [2, 3]. In this study, treatment with CoQ10 demonstrated improvement in renal hemodynamics with reduced RVR and elevated RBF. Studies suggest that CoQ10 stimulates the production of prostaglandin-1 and prostacyclin, which aid vasodilation and reduce peripheral resistance by preserving the vasodilator nitric oxide, promoting the reduction of nitrogen dioxide to nitric oxide, helping to maintain this bioregulatory agent [11, 13].

In the present study a significant increase of oxidative stress via urinary TBARS, peroxides, and nitrate elevation and reduction of thiols in renal tissue was observed. Hyperglycemia increases oxidative stress in CI-AKI by activation of stress-activated protein kinases, functional proteins glycosylation, glucose autoxidation and the formation of reactive nitrogen species, such as peroxynitrite, that has been related to enhanced inflammation in diabetes by decreased nitric oxide bioavailability [10, 18, 33]. ROS production in DM has been linked to vasoconstriction, vascular cell hypertrophy and migration, endothelial dysfunction, modification of extracellular matrix proteins, and increased renal sodium reabsorption [3, 33]. Enhanced macrophage migration induces the release of inflammatory and profibrotic cytokines, stimulating greater ROS production. Thus, the oxidative stress induced by cytokine production in DM associated with contrast injury increases ROS, creating a vicious cycle [2, 34–36].

Despite its primary role in the production of ATP, CoQ10 is considered a substance with great antioxidant and anti-inflammatory properties, due to its capability of stabilizing two free radicals to each molecule of CoQ10 in its redox cycle; its quick recovery, the ability to inhibit protein kinases and reduce NF-kB levels; reduction of free radicals by using them to the recovery of antioxidant cycle; its efficiency in interrupting radical chain reactions such as lipid peroxidation, and its ability to avoid nitrosative stress [37, 38].

In this study, the treatment of diabetic animals with CoQ10 demonstrated the ability to maintain the reserve of renal thiols antioxidant after IC administration. Intracellular antioxidants mechanisms such as glutathione, a non-protein thiol, take part in the neutralization of ROS, protecting against oxidative damage. CoQ10 demonstrated to preserve glutathione in animal models with cisplatin-induced nephrotoxicity by the increase of selenium, necessary for the composition of glutathione [31, 39, 40].

In the present study, diabetic animals demonstrated mild tubulointerstitial changes typically seen in the development of diabetic nephropathy [41]. The histological changes observed in animals that received IC were due to the association of the insult caused by hyperglycemia and IC, reinforcing that the mechanism involved in the reduction of renal function is mainly related to renal hemodynamic changes and oxidative damage, which favor the installation of CI-AKI. Our findings indicate that the administration of CoQ10 prevented the progression of the extension area with tissue damage after the use of IC.

Considering that DM is a modifiable risk factor for IC nephrotoxicity, the implementation of preventive strategies with innovative pharmacological interventions, such as CoQ10, can establish a promising scenario and reverse the negative effects of pathophysiology of the CI-AKI.

## Conclusion

Corroborating with the findings of Chen et al. that demonstrated the protective effect of CoQ10 in combination with trimetazidine against CI-AKI in patients with coronary heart disease complicated with renal dysfunction as well as on rat models of CI-AKI, the results of this study have demonstrated the renoprotection of CoQ10 in an experimental model of risk factor of DM for CI-AKI [42]. In the present study, CoQ10 presented an antioxidant effect on the CI-AKI in male diabetic rats by improving renal function and renal hemodynamics, preserving morphology and reducing oxidative stress.

## Data Availability

The datasets during and/or analysed during the current study available from the corresponding author on reasonable request.

## Abbreviations

DM: Diabetes mellitus
CI-AKI: Contrast-induced acute kidney injury
ROS: Reactive oxygen species
CoQ10: Coenzyme Q10
CEEA: Committee of Experimental Animals, University of Sao Paulo
STZ: Streptozotocin
IC: Iodinated contrast
i.p.: Intraperitoneally
i.v.: Intravenous
DM + IC: Diabetes mellitus + iodinated contrast
DM + IC + CoQ10: Diabetes mellitus + iodinated contrast + coenzyme Q10
Cr: Creatinine
RBF: Renal blood flow
RVR: Renal vascular resistance
TBARS: Urinary thiobarbituric acid reactive substances
TCA: Trichloroacetic acid
SEM: Standard error
